# Misophonia reactions in the general population are correlated with strong emotional reactions to other everyday sensory–emotional experiences

**DOI:** 10.1098/rstb.2023.0253

**Published:** 2024-08-26

**Authors:** Solena D. Mednicoff, Sivan Barashy, David J. Vollweiler, Stephen D. Benning, Joel S. Snyder, Erin E. Hannon

**Affiliations:** Department of Psychology, University of Nevada, Las Vegas, NV 89154-9900, USA

**Keywords:** misophonia, autonomous sensory meridian response (ASMR), musical chills, frisson, musicality

## Abstract

Misophonic experiences are common in the general population, and they may shed light on everyday emotional reactions to multi-modal stimuli. We performed an online study of a non-clinical sample to understand the extent to which adults who have misophonic reactions are generally reactive to a range of audio-visual emotion-inducing stimuli. We also hypothesized that musicality might be predictive of one's emotional reactions to these stimuli because music is an activity that involves strong connections between sensory processing and meaningful emotional experiences. Participants completed self-report scales of misophonia and musicality. They also watched videos meant to induce misophonia, autonomous sensory meridian response (ASMR) and musical chills, and were asked to click a button whenever they had any emotional reaction to the video. They also rated the emotional valence and arousal of each video. Reactions to misophonia videos were predicted by reactions to ASMR and chills videos, which could indicate that the frequency with which individuals experience emotional responses varies similarly across both negative and positive emotional contexts. Musicality scores were not correlated with measures of misophonia. These findings could reflect a general phenotype of stronger emotional reactivity to meaningful sensory inputs.

This article is part of the theme issue ‘Sensing and feeling: an integrative approach to sensory processing and emotional experience’.

## 1. Background

Misophonia is a disorder characterized by negative emotional reactions to every-day sounds like chewing, tapping, breathing and other human-produced and animal-produced actions [1–3]. The recent consensus definition for misophonia classifies the disorder as a decreased tolerance to specific sounds and separates misophonia from other decreased sound tolerance disorders like hyperacusis and phonophobia, as it is the context or meaning of the sound and not the loudness that elicits a misophonic response [3]. It is for this reason, for example, that a misophonic sound can be made less aversive when paired with visual information that suggests it emanates from a less aversive sound-producing object [4,5]. Similarly, higher-level personality and emotional traits are correlated with misophonia, such as disgust sensitivity, emotional dysregulation, neuroticism, anxiety, depression, obsessiveness and personality disorder symptoms [6–11]. These findings underscore that misophonia is much more than a sensory-processing disorder and may be related to abnormalities in affective processing that lead individuals with misophonia to have aversive emotional reactions to stimuli that others do not find particularly bothersome.

Consistent with the idea that misophonia is not purely a sensory-processing disorder, early clinical descriptions of misophonia point out that misophonia occurs without any apparent low-level hearing impairment [1,2]. Undergraduates with moderate misophonia had normal pure-tone thresholds and normal latencies and amplitudes of auditory brainstem event-related potentials (ERPs) compared to those with minimal misophonia symptoms [12]. Similarly, misophonia was not correlated with pure-tone hearing thresholds nor with between-ear differences in hearing thresholds or uncomfortable loudness, implicating a central nervous system mechanism for misophonia [13].

In contrast to the apparently intact peripheral processing in misophonia, higher-level auditory abnormalities in those with misophonia may shed light on the mechanisms of the misophonic experience. Auditory cortical N1 ERPs in response to oddball/infrequent tones were smaller for patients with misophonia than for a control group but were no different for standard/frequent tones [14]. Given the overlapping timing of the N1 (which can peak as late as 120 ms) and the mismatch negativity (MMN, which tends to peak at 150 ms) [15], this finding could reflect an abnormally small MMN in people with misophonia. Evidence from functional magnetic resonance imaging (fMRI) suggests that compared to those without misophonia, patients with misophonia have increased activation of the auditory cortex, anterior insula and anterior cingulate cortex when presented with misophonia triggers [16]. There is also evidence for central nervous system abnormalities in misophonia, such as increased fMRI activation of posterior cingulate cortex, superior medial frontal cortex and dorsolateral prefrontal cortex during a response inhibition task [17]; activation of auditory cortex, insula, primary motor cortex, premotor cortex, prefrontal cortex, hippocampus and cerebellum while listening to misophonia triggers [18–20]; and functional connectivity patterns of insula, auditory cortex, visual cortex and ventral premotor cortex with a variety of other areas [19–21]. Thus, studies of brain activity implicate a wide range of cortical areas depending on the task, underscoring the importance of high-level affective processing and emotion regulation mechanisms in misophonia, consistent with behavioural findings discussed above.

Increasing evidence suggests that the experience of misophonia is widespread among those not clinically diagnosed with the disorder. Depending on the sample size, form of recruitment and measure, the self-reported prevalence of misophonia has been estimated to range from 6 to 55% [22–26]. Studies of non-clinical samples in multiple languages suggest that mild misophonic experiences are quite common in the general population throughout the world [13,25–30]. Less is known about the relationship between misophonia and other high-level affective auditory experiences in the general population, particularly those that evoke positive emotional responses such as autonomous sensory meridian response (ASMR) videos and musical chills [31].

Like misophonia, ASMR entails a combination of complex emotional responses that are driven by meaningful audio-visual stimuli. ASMR is a relaxed state in which individuals experience tingling, typically across the back of the head and shoulders, in response to certain stimuli. However, unlike misophonia, these responses are described as relaxing and positive, often accompanied by tingling that starts in the head and moves down towards the extremities [31–33]. Common ASMR triggers overlap with misophonia triggers like chewing, breathing and tapping, but they include others that are more unique to ASMR like origami folding, hair brushing and pouring of liquid [34]. ASMR and misophonia share some triggers, and people who tend to experience ASMR are more likely to experience misophonia than those who do not experience ASMR [35,36]. There is also evidence for overlap in personality traits associated with both ASMR and misophonia, such as neuroticism [37–39] and schizotypal traits [40,41].

Both ASMR and musical chills are positive valence experiences that can include tingling sensations and the two experiences have yet to be fully disentangled and distinguished from one another [35]. Musical chills are typically evoked by emotionally intense moments in music rather than environmental sounds [34,42–44]. Most listeners report experiencing chills when there are surprising or unexpected events, such as the entrance of a new voice, unexpected changes in instrumentation, loudness, number of events, melody, harmony, key or an event occurring earlier or later than expected [45–48]. Because musical ‘surprises’ can only occur if a listener has robust expectations about the way familiar genres of music are typically structured, musical knowledge and skills may play a key role in an individual listener's likelihood of experiencing chills [46].

A general question of interest is whether musical expertise or musicality plays a role in auditory affective phenomena such as chills, ASMR and misophonia. While some studies report that musicians experience chills more often than non-musicians [49–51], most listeners—regardless of music training—can recognize emotions conveyed in music [52–55], and as many as 90% of listeners report experiencing musical chills sometimes [44,45,50]. These results are consistent with the finding that music aptitude and engagement vary widely in the untrained population and predict emotional processing both within and outside the context of music [56–58]. The tendency to experience musical chills may relate to more general emotional processes relevant to ASMR and misophonia. Indeed, some studies report weak to moderate correlations between ASMR and chills propensity [59], and both ASMR and musical chills are correlated with openness to experience [39,50,60–62]. Although there are anecdotal reports of musicians experiencing misophonia [63], evidence for a relationship between misophonia and musicality is mixed, with some studies reporting no association [64] and others reporting less misophonia in musicians than in non-musicians [65]. While some have suggested potential links between misophonia, ASMR, musical chills and emotional processing [66], to our knowledge, no studies have examined the relationship between misophonia and musical chills.

Overlapping brain areas appear to be involved in misophonia, ASMR and musical chills, as suggested by fMRI studies showing activation of such areas as the insula, ventral striatum and cingulate cortex across these phenomena [17–20,67–71]. Furthermore, people who experience ASMR have greater interoceptive sensitivity and body awareness [72], and overlapping parts of the insula are active while judging one's own heartbeat and judging one's emotional reactions to videos [73]. Heartbeat-evoked potentials—which arise from the insula as a hub of interoceptive processing [74–76]—are larger for people who experience ASMR frequently than for people who experience ASMR infrequently [77], although this study failed to find group differences on behavioural measures of interoceptive awareness.

To address the limitations in our knowledge about misophonia in the general population and how it relates to ASMR, musical chills and musicality, we conducted an online study of around 300 adult participants. We decided to focus on the general population because less is known about misophonia reactions in people with sub-clinical levels of misophonia and because of the importance of understanding auditory emotional reactions in all people, not just those with a diagnosable disorder. Participants filled out a misophonia self-report scale, questions about ASMR and a musical sophistication scale with a sub-factor measuring emotional engagement with music [78,79]. Participants watched videos intended to evoke misophonia, ASMR, musical chills, annoyance or neutral feelings, and reported in real time any emotional reactions they experienced by pressing a button; participants also provided arousal and valence ratings after each video. The goal of this approach was to examine how individual responses might vary across these different phenomena. We hypothesized that people who tend to have stronger misophonic reactions might also tend to have stronger emotional reactions of other types, such as ASMR and musical chills, and might also have stronger emotional engagement with music.

## 2. Methods

### (a) Participants

All methods were approved by the University of Nevada, Las Vegas (UNLV) Institutional Review Board. A total of 301 people were initially recruited for this study from October 2021 to July 2023 (see table 1 for demographic information regarding age, sex, etc.). Participants were eligible to take the study if they self-reported that they were at least 18 years of age, spoke English proficiently, were healthy (e.g. no cold or ear infections that could interfere with hearing audio stimuli) and had normal-to-corrected hearing and vision. We excluded participants who failed attention and headphone/quiet testing location checks (*n* = 16) or who had difficulty loading most or all of the sounds (*n* = 7) (see §2c below), resulting in a final sample of 278 participants (table 1). Participants were recruited from the UNLV Psychology Participant Pool (20.9%), university listservers (9%), snowball sampling with family and friends of the lab (21.6%), or advertisements posted on personal and lab social media pages (2.5%), Reddit pages (39.2%), Facebook groups (5.8%) and misophonia support/advocacy groups (1%). Only participants from the UNLV Psychology Participant Pool were compensated with course credit; all other participants were volunteers who received no compensation.

**Table 1.** Participant demographics.

	full sample	
	***n***	**%**
**age (years)**		
mean	30.7	—
standard deviation	13.3	—
range	18–76	—
**gender**		
male	93	33.5
female	166	59.7
non-binary	7	2.5
gender-fluid	3	1.1
agender	4	1.4
other	2	0.7
prefer not to respond	3	1.1
**current country**		
United States	209	75.2
Canada	23	8.3
other	46	16.5
**native language**		
English	213	76.6
other	65	23.4
**length of formal musical training (years)**		
0–1	131	47.1
2–3	84	30.2
4+	63	22.7

Two different versions of advertisements were posted on social media pages. The first, general advertisement described the study as investigating emotional responses to meaningful sounds in the world. The second specifically mentioned misophonia, ASMR and musical chills and was only posted to Reddit pages or Facebook groups directly related to these experiences. We used these two sets of advertisements to enhance the likelihood we would sample from a broad range of participants who did or did not experience misophonia, ASMR and musical chills. When given the opportunity to share any other potentially relevant information about themselves (an open-ended question), some participants spontaneously shared that they had synesthesia (*n* = 2), tinnitus (*n* = 1), autism spectrum disorder (*n* = 3), obsessive–compulsive disorder (OCD; *n* = 1), anxiety (*n* = 1) or attention-deficit/hyperactivity disorder (ADHD; *n* = 6).

### (b) Apparatus and procedure

This study was implemented online using the online survey builder Qualtrics (Qualtrics, Provo, UT; https://www.qualtrics.com/). Participants were asked to wear headphones, set their volume to a comfortable listening level and use a tablet, laptop or computer in a quiet environment free from distractions. No mobile phones were allowed.

Upon clicking on the Qualtrics survey link, participants were required to consent before taking part in this study. The study began with a headphone check, followed by separate misophonia, ASMR and musical chills blocks, presented in a random order. Each of these blocks first presented a definition of the phenomenon (misophonia, ASMR or chills), self-report questions and a video reactions task. Next, participants completed the Goldsmiths Musical Sophistication Index (Gold-MSI; see §2g below) and answered additional background questions. Finally, participants completed attention checks and follow-up questions about recruitment, feedback on the study and future research participation.

### (c) Headphone and attention checks

At the start of the study, participants completed the headphone check from Woods *et al*. [80] by judging which of three pure tones was the quietest across six trials, with one of the tones presented with a 180° phase difference across the two channels and thus noticeably quieter only if the participant wore headphones [80]. To pass the headphone check, participants had to get 5 out of 6 trials correct. However, we did not exclude participants who failed the headphone check if they reported testing in a quiet location, as described in the following paragraph since we were most concerned with whether they could clearly hear the sounds, either over headphones or in free field.

To ensure optimal quality of online data collection, we asked several additional questions to assess whether participants were paying attention, following instructions and performing the task in an appropriate environment [81,82]. Throughout the study, we probed participants with four attention check questions that instructed them to respond incorrectly, on purpose (e.g. ‘What colour is the sky? Please answer this incorrectly, on purpose, by choosing YELLOW instead of blue’). At the end of the study, we asked participants about their testing environment (how noisy?), whether they wore headphones and whether or not they had trouble loading videos and sounds. Finally, we asked them to tell us how careful they were ('How carefully did you complete the study? Please answer honestly’), with five response options from ‘not at all carefully’ to ‘very carefully’. Because we wanted to maximize the likelihood that sounds in the study were audible, we excluded any participants who did the study in a noisy environment *and* failed the headphone check. If participants were in a quiet environment, we did not exclude them for not wearing headphones. Because we could not verify the quality of the internet connection, we also excluded participants who indicated they had trouble loading most or all of the sounds. Similarly, we attempted to flag participants who were largely non-compliant by excluding any who incorrectly answered more than two attention check questions or who answered that they did not take the survey carefully at all.

### (d) Misophonia self-report

Before answering any survey questions about misophonia specifically, participants were provided with the following definition of misophonia: ‘Misophonia is a condition characterized by the experience of strong negative reactions (e.g. anger and anxiety) to everyday (trigger) sounds, such as eating, breathing, tapping, etc’.^1^ Participants then completed the Amsterdam Misophonia Scale (A-MISO-S), which presents six items, each yielding a score from 0–4 that contributes to an overall misophonia severity score [84]. Scored items ask about (1) the amount of time spent occupied by misophonic sounds; (2) misophonia's interference with social functioning; (3) level of distress and anger towards misophonic sounds; (4) effort to resist thinking about the misophonic sounds; (5) control over thoughts and anger about the sounds; and (6) time spent avoiding misophonic situations. According to Schröder *et al*. [84], overall scores are interpreted as follows: 0–4 subclinical (*n* = 91), 5–9 mild (*n* = 95), 10–14 moderate (*n* = 72), 15–19 severe (*n* = 17) and 20–24 extreme (*n* = 3) (see the electronic supplementary material, figure S1 for a histogram of these numbers). We used the overall A-MISO-S score as a measure of misophonia severity.

After completing the A-MISO-S, participants were presented with a list of 26 common triggers and asked to select all sounds that bothered them, followed by an open-ended prompt to list any other bothersome sounds. Participants also self-reported the age at which they first experienced stress to these sounds, as well as the emotions that the triggering sounds elicit (by choosing from response options of irritation, anger, fear, disgust or open-ended response). Table S1 in the electronic supplementary material lists and shows the frequencies with which participants selected each of the 26 common triggers, as well as the 5 options for the emotions that the triggers elicited.

### (e) ASMR and chills self-report

We provided definitions of ASMR and chills to participants before they answered questions about each experience. We defined ASMR as ‘a relaxed state in which individuals experience tingling, typically across the back of the head and shoulders, in response to certain stimuli. These stimuli often (but do not necessarily) include whispering, crisp noises (such as tinfoil), and watching others do simple repetitive tasks. ASMR is not frisson (e.g. musical chills when listening to music).’ Participants were asked whether or not they experience ASMR (yes, no, unsure), whether ASMR relaxes them, and whether they purposefully search for ASMR videos.

Musical chills were defined as follows: ‘When listening to music, some people experience musical chills (e.g. frisson), which are often described as ‘a rapidly spreading, tingling feeling’ often accompanied by raised hairs and goosebumps. These feelings of a tingle or shudder might be physical, emotional, or both’. The participant was then asked if he/she experiences musical chills (yes, no, unsure).

### (f) Video reactions task

Participants were presented with a total of 37 videos across three blocks of testing. Ten 15Cs-long videos were presented for each of the misophonia and ASMR blocks, and five 60 s music videos were presented in the musical chills block. Musical videos were longer than the vidoes for all other conditions because we wanted to maximize the likelihood of participants experiencing chills, given that musical chills often arise over prolonged music listening and take time to unfold [85]. In addition, ten 15 s ‘neutral’ videos were randomly interspersed throughout the three blocks, as well as two 15 s videos intended to be ‘annoying’ for most participants regardless of misophonia. See electronic supplementary material, table S2 for the list of videos, and the following link to view the videos: https://osf.io/k6h2m/. Along with asking participants to set their volume to a comfortable listening level, all videos were calibrated within Qualtrics to be at an equal volume.

Videos were selected by six people using an iterative stimulus selection process to find, select and edit YouTube videos. This small listening group consisted of young adults who frequently use the Internet and were familiar with and experienced these phenomena. For each candidate video, listeners provided ratings of the extent to which the video elicited the desired response (misophonia, ASMR or chills), as well as the emotional valence of the video. ASMR and misophonia videos were chosen based on common triggers in the literature [32,66,83,86]. Prior studies of musical chills have used a range of classical, popular, film and live music recordings, often self-selected by the participant and typically presented in the auditory modality [46]. Because we wanted to present videos across all conditions, we perused Internet forums listing songs that elicit chills and found videos of live performances for each song. We then selected from among these videos the five videos that elicited the most chills among our small listener group. We also selected a set of environmental videos that the listening group rated as emotionally neutral, such as scenes from a campfire, a beach, or a thunderstorm (see electronic supplementary material, table S1). Finally, two videos (baby crying, fingernails on a chalkboard) were selected that were expected to be aversive to all participants, regardless of misophonia [87,88].

**Figure 1.**
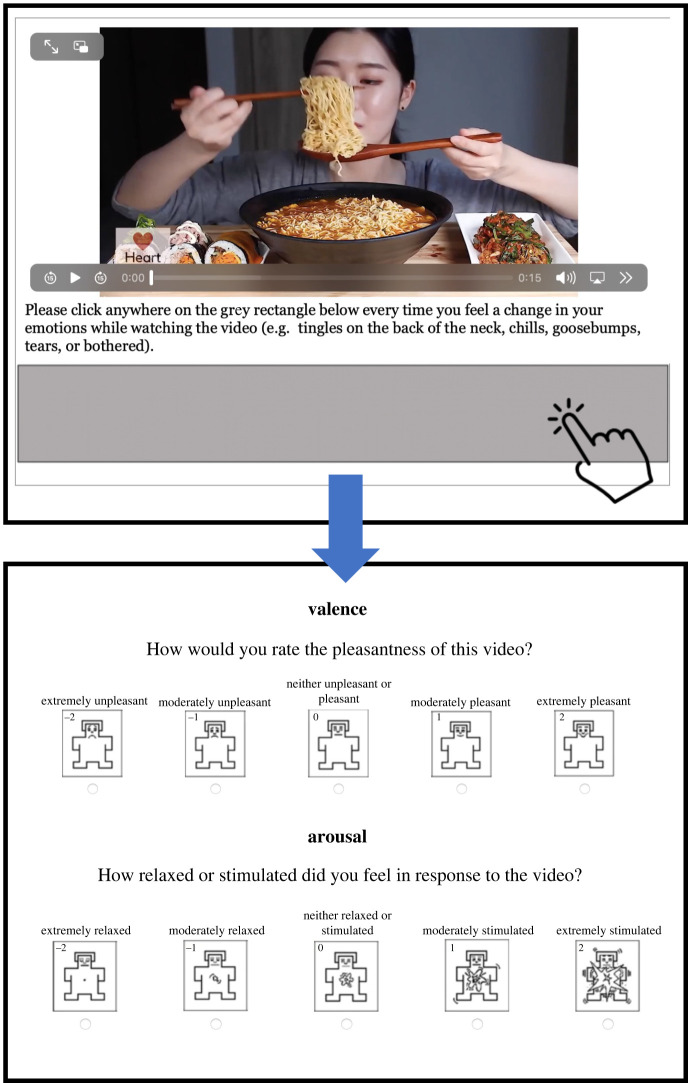
Schematic of the video reactions task showing what a participant sees on a trial, beginning with the reactions prompt with video (top), followed by valence and arousal ratings (bottom).

Videos played automatically on each trial, but participants could immediately advance to the next trial if a particular video was too upsetting. At the beginning of the 'video reactions task' for each block, participants were given a reminder definition of each phenomenon (misophonia, ASMR or musical chills). While watching videos, participants were instructed to click on a grey box positioned below the video each time they felt any emotional reaction to the video. These reactions could be tingles on the back of the neck, chills, goosebumps, tears, or feelings of irritation, disgust, pleasure or relaxation. Thus, for each video, we obtained a measure of ‘reactions’ that reflected the number of times a participant pressed the button during each 15 second video (for musical chills videos, this number was divided by 4 to adjust for the longer duration of music videos). After the video ended, we used the self-assessment manikin (SAM) [89] to prompt participants to provide a rating of valence ('How would you rate the pleasantness of this video?') and arousal ('How relaxed or stimulated did you feel in response to the video?'), with five response options from ‘extremely unpleasant/relaxed’ to ‘extremely pleasant/stimulated’, scored later from −2 to +2 (see figure 1 for a schematic of a trial). Although a 9-point scale is often used with the SAM, we opted to use the 5-point version because it yields similar results but is more accessible to those with low literacy skills [90]. This task thus yielded for each video a measure of a participant's emotional reactions (total clicks during the video), as well as the participant's valence and arousal ratings for that video.

### (g) Musicality and background measures

Musicality was measured using the Gold-MSI [79], which is a 39-item questionnaire measuring five factors of musical sophistication: active engagement, perceptual abilities, musical training, singing abilities and emotional engagement. For our analyses, we used the overall Gold-MSI score (*ω* = 0.94), the musical training sub-factor (*ω* = 0.91) and the emotional engagement sub-factor (*ω* = 0.77). We selected these two sub-factors because we were interested in addressing whether musicians are more likely to experience misophonia and to address our hypothesis that misophonia might entail stronger emotional reactions and engagement to music. The omega (*ω*) values listed above are higher than 0.70, which indicates acceptable internal consistency [91]. Immediately after the Gold-MSI questions, we asked demographic questions about age, gender, ethnicity, current country of residence, language background, country of residence during childhood/adolescence, current occupational status, highest education acquired, parents' educational background, number of languages spoken, music habits (such as how often the participants listened to music or played an instrument) and family members who were musicians.

### (h) Data analysis

Data are available at this link: https://osf.io/k6h2m/. All analyses were conducted using JASP (v. 0.17.3; see https://jasp-stats.org/). A Bayes factor (*BF*) in support of the null hypothesis (*BF*_01_) or the non-null hypothesis (*BF*_10_) is reported for each analysis and interpreted as follows: *BF* > 1 is ‘anecdotal evidence’; *BF* > 3 is ‘some evidence’; *BF* > 10 is ‘strong evidence’ and *BF* > 30 is ‘very strong evidence’ [92,93]. Importantly, *BF*_10_ and *BF*_01_ are simply inverses of each other, i.e. *BF*_10_ = 1/*BF*_01_, such that when a very small *BF*_10_ is shown in one of our tables, this implies that the *BF*_01_ would be fairly large. Pearson's correlations (using a standard Cauchy prior width = 1.0 to specify the expected plausible range effects before observing the data, alternative hypothesis = correlated) were calculated along with an accompanying *BF*_01_ or *BF*_10_ for the following variables: A-MISO-S scores, reactions, valence and arousal for each video type (misophonia, ASMR and musical chills) and the overall score for Gold-MSI, for the 'music training' sub-score, and the 'emotional engagement' sub-score. The use of Bayesian statistics was chosen over frequentist statistics for this study in part because of the exploratory nature of the study and because our interest was in evaluating the strength of both non-null and null findings using *BF*_10_ and *BF*_01_, respectively. The *BF* range represents the strength of evidence for the alternative hypothesis relative to the null, which allows us to avoid dichotomized decisions (such as *p*-values in frequentist statistics) to accept/reject the hypothesis [94]. Correction for multiple comparisons (e.g. the simple correlations reported below) was deemed not necessary, consistent with recommendations for Bayesian analysis [95].

Three Bayesian regressions were conducted using misophonia reactions, ASMR reactions and chills reactions as dependent variables, respectively. Predictors were A-MISO-S, the valence and arousal for the corresponding type of video and the reactions for the other two types of videos. For example, the regression predicting misophonia reactions used A-MISO-S, valence and arousal ratings for the misophonia videos, and ASMR and chills video reactions as predictor variables. Comparisons were made to the null model and the preferred model was the one with the highest *BF*_10_ value, regardless of *R*^2^ value. The best 10 models are displayed in the regression tables. We used the Jeffreys-Zellner-Siow Prior (JZS) with *r*-scale = 0.354 to assign a normal distribution to each regression coefficient [96], and the model prior was beta binomial with parameters *a* = 1 and *b* = 1, which is a flat distribution. We used the sampling method of Bayesian adaptive sampling (BAS), which samples models without replacement [97], with number of models set to the default 0, and the number of samples for credible interval calculation = 1000. Setting the number of models to 0 means the analysis will attempt to enumerate all models. These are all default parameters in JASP.

## 3. Results

### (a) Planned analysis: correlations

**Figure 2.**
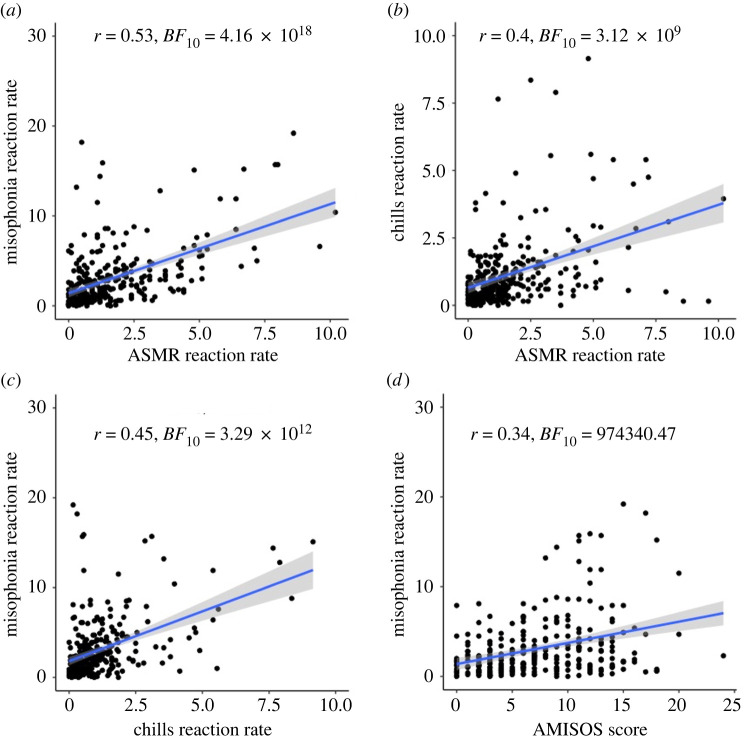
Scatterplots for A-MISO-S, and reaction rates (number of clicks per 15 s) to misophonia, chills and ASMR videos. (*a*) ASMR and misophonia reaction rate correlation. (*b*) ASMR and chills reaction rate correlation. (*c*) Chills and misophonia reaction rate correlation. (*d*) A-MISO-S and misophonia reaction rate correlation. The reaction rates are mean numbers of reactions for each participant in each video category, normalized by the duration of the videos to control for differences in video length across the video categories.

Misophonia reactions were correlated with A-MISO-S scores, suggesting that real-time reactions to misophonia triggers are related to self-reported misophonia severity (figure 2 and electronic supplementary material, table S3). Misophonia reactions, ASMR reactions and chills reactions were all correlated moderately with each other (see figure 2 for scatterplots), providing initial evidence that people who have relatively frequent misophonia reactions tend to have frequent ASMR (*r*_276_ = .53, *BF*_10_ *=* 4.16 × 10^18^) and chills reactions (*r*_276_ = 0.45, *BF*_10_ *=* 3.29 × 10^12^). Consistent with this finding, A-MISO-S scores were also correlated with ASMR reactions (*r*_276_ = 0.26, *BF*_10_ *=* 1145.60) but less so with chills (*r*­ = 0.16, *BF*_10_ *=* 3.12). Misophonia reactions were correlated negatively with misophonia valence (*r*_276_ = −0.31, *BF*_10_ *=* 74 070.18), and positively with misophonia arousal (*r*_276_ = 0.35, *BF*_10_ *=* 4.09 × 10^6^), demonstrating that participants who had more frequent reactions to misophonia videos also gave those videos more negative evaluations and higher arousal ratings. A-MISO-S scores showed a similar pattern of correlation with misophonia valence (*r*_276_ = −0.28*,* *BF*_10_ *=* 4,988.95) and arousal (*r*_276_ = 0.41, *BF*_10_ *=* 5.66 × 10^9^). One potential concern with the reactions measure is that it could simply reflect a generic propensity to press the button, rather than truly reflecting the degree of emotional reactivity. Such a propensity could explain high correlations between misophonia, ASMR and musical chills reactions; however, the pattern of correlations in electronic supplementary material, table S3 suggests that this is probably not the case, given that reactions do not correlate with arousal or valence scores for ASMR and musical chills videos.

ASMR reactions were negatively predicted by ASMR valence (*r*_276_ = −0.19, *BF*_10_ *=* 10.12) and positively predicted by ASMR arousal (*r*_276_ = 0.32, *BF*_10_ = 227 914.89), which stands in contrast to reports in the literature that ASMR is usually experienced as pleasant and relaxing [32–34]. These results suggest that at least some participants in our sample had misophonic reactions to some or all ASMR videos, a possibility we explore below (Exploratory Analysis).

As shown in electronic supplementary material, table S4, neither the overall Gold-MSI scores, the music training sub-scores nor the emotional engagement sub-scores were correlated above *r* = 0.12 with A-MISO-S or reactions, valence and arousal to misophonia or ASMR videos. Many of the correlations were much smaller than *r* = 0.10, with some *BF*_01_ values > 10, i.e. supporting the null hypothesis that there is no relationship between these measures and Gold-MSI scores. Valence of chills was correlated with emotional engagement; however, overall Gold-MSI and music training were not correlated with any chills variables, consistent with the notion that greater music training or sophistication does not necessarily lead to more musical chills [79]. Consequently, none of the Gold-MSI scores were used in the following regressions.

### (b) Planned analysis: regressions

Valence ratings for all video conditions for all participants can be seen in electronic supplementary material, table S2. Electronic supplementary material, tables S5–S10 show which sets of regression predictors resulted in the best *R*^2^ values and *BF*_10_ values for the three dependent variables: misophonia video reactions, ASMR video reactions and chills video reactions. The highest *BF*_10_ value predicting the misophonia video reactions (electronic supplementary material, table S5) resulted from the model that included misophonia valence, ASMR reactions, chills reactions and A-MISO-S as predictors (see electronic supplementary material, table S6 for predictor coefficient estimates), which only had a slightly lower *R*^2^ than the full model. The highest *BF*_10_ value for the ASMR video reactions (electronic supplementary material, table S7) resulted from the model that included ASMR arousal, misophonia reactions and chills reactions (see electronic supplementary material, table S8 for predictor coefficient estimates), which only had a slightly lower *R*^2^ than the full model. The best *BF*_10_ value for the chills video reactions (electronic supplementary material, table S9) was for the model that included chills arousal, misophonia reactions and ASMR reactions (see electronic supplementary material, table S10 for predictor coefficient estimates), which only had a slightly lower *R*^2^ than the full model.

In summary, for all three video types, the number of reactions across participants was generally best predicted by reactions to the other two video types and arousal to the video type being predicted. For misophonia video reactions, the best model also included misophonia video valence and A-MISO-S scores, but A-MISO-S was not a strong predictor for the ASMR and chills reactions regressions. The fact that reactions to any one video type were predicted by reactions to the other video types supports the notion that some people tend to have more emotional reactions while watching videos as a general characteristic, across both positively and negatively valenced experiences.

### (c) Exploratory analysis: recoded data regressions

**Figure 3.**
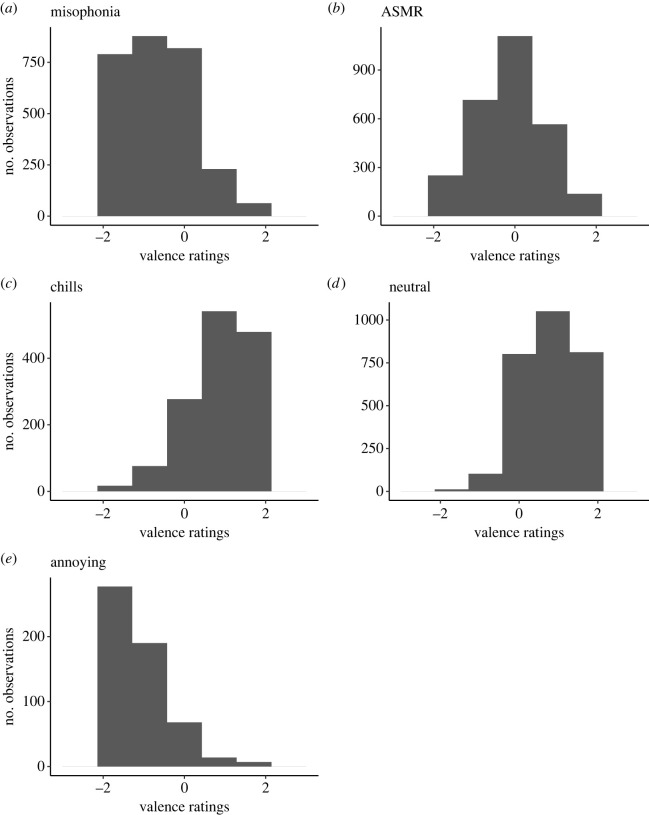
Histograms showing distribution of valence values for misophonia (*a*), ASMR (*b*), chills (*c*), neutral (*d*) and annoying (*e*) videos.

As noted in §1, ASMR videos sometimes give rise to misophonia reactions. To see if this was the case in the present study and whether the analyses above were mixing ASMR and misophonia responses within the same regression, we plotted distributions of valence for the three video conditions to check if misophonia videos were generally experienced negatively and ASMR videos were generally experienced positively. Histograms of valence ratings for all video conditions are shown in figure 3. Participants' ratings for misophonia videos were mostly negative, as intended, with only a few ratings near or above zero (*M* = −0.76). Despite the expectation that ASMR videos would elicit positive emotions, participants’ ratings for this type of video were slightly negative on average (*M* = −0.14). Musical chills videos were mostly experienced with positive valence (*M* = 1.0 for frisson), as expected. Surprisingly, neutral videos were experienced with mostly positive valence (*M* = 0.92). Finally, annoying videos were experienced with negative valence (*M* = −1.29), as expected.

We recoded our video reactions task data according to valence, as described below, to obtain purer measures of misophonia and ASMR experiences. We did not recode responses to musical chills videos because chills are considered a phenomenon distinct from ASMR, even though both are usually positive experiences [43,98]. Likewise, we did not recode annoying videos because misophonia reactions are thought to be distinct from reactions to general sounds that annoy everyone, like babies crying and nails on a chalkboard [87], and valence ratings of these videos confirmed that this was the case (see electronic supplementary material, table S1). However, we recoded the neutral videos because some of them included sounds like wind blowing, fire and water sounds that are often described as potential ASMR triggers [34,99]. Electronic supplementary material, table S2 provides detailed information about the 37 videos, including mean valence and arousal for each video and correlations between the video reactions task measures.

For this exploratory analysis, we recoded misophonia, ASMR and neutral videoswhose valence ratings were not as expectedto create two new valence-based categories of reactions for each participant. Specifically, any video that was rated by a particular participant as having a valence of −2 or −1 was considered ‘negative’ and any video with a valence rating of 1 or 2 was considered ‘positive,’ and mean reactions and arousal ratings were re-calculated for the new positive and negative video categories. Videos with valence ratings of 0 were not included in these means. After recoding, there were 2860 positive valence observations and 2750 negative valence observations, suggesting we had comparable observations of both positive and negative valence categories. We then performed correlations using the new variables, and two Bayesian regressions, one predicting reactions to negative valence videos and one predicting reactions to positive valence videos. Predictors for these regressions included A-MISO-S, reactions to the opposite valence videos, reactions to the chills videos and arousal for the corresponding valence videos. We used the newly created negative and positive reactions as dependent variables in new regressions to mirror our original set of analyses that sought to understand misophonia, ASMR and chills reactions, respectively.

**Figure 4.**
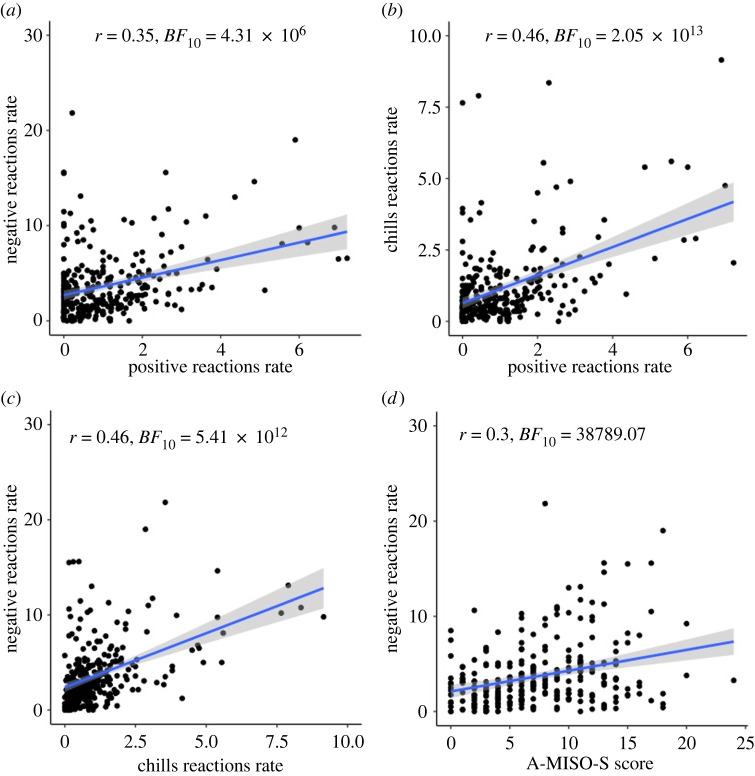
Scatterplots for A-MISO-S, and reactions to negative and positive valence videos. (*a*) Positive and negative reactions correlation. (*b*) Positive and chills reactions correlation. (*c*) Chills and negative reactions correlation. (*d*) A-MISO-S and negative reactions correlation. The reaction rates are mean numbers of reactions for each participant in each video category, normalized by the duration of the videos to control for differences in video length across the video categories.

As shown in electronic supplementary material, table S11 and figure 4, A-MISO-S scores were correlated strongly with reactions and arousal ratings for negative valence videos but less so for reactions and arousal ratings for the positive valence videos. Similarly, reactions and arousal ratings were correlated only for the same valence category (e.g. reactions to negative valence videos were correlated with arousal to negative valence videos but not with arousal to positive valence videos). This result is consistent with our interpretation that the recoded valence categories more purely reflect misophonic versus non-misophonic reactions. Nevertheless, negative and positive reactions were still correlated with each other, as before. Reactions or arousal to recoded video categories were again not correlated with Gold-MSI scores (electronic supplementary material, table S12).

Electronic supplementary material, table S13 shows that the best *BF*_10_ value for the reactions to negative valence videos resulted from the full model (see electronic supplementary material, table S14 for predictor coefficient estimates). Electronic supplementary material, table S15 shows that the best *BF*_10_ value for reactions to positive valence videos resulted from the model that included arousal to positive valence videos, reactions to negative valence videos and chills reactions (see electronic supplementary material, table S16 for predictor coefficient estimates), which had the same *R*^2^ value as the full model. These findings are reminiscent of our planned regressions, with same-valence arousal and reactions to other types of videos consistently predicting reactions for both negative and positive valence videos. Given that we believe these two types of reactions more accurately reflect misophonia and ASMR reactions, respectively, we believe this analysis further solidifies our original interpretation, namely that some people are more emotionally reactive, regardless of the type of emotional experience.

## 4. Discussion

Our non-clinical sample of participants completed self-reports of misophonia and musicality and then indicated their emotional responses to several types of videos. Our main finding was that emotional reactions to misophonia, ASMR and musical chills videos were all correlated. This pattern remained even after re-categorizing reactions to misophonia, ASMR and neutral videos based on each participant's valence ratings for each video to increase the likelihood that negative and positive video categories gave rise to misophonia and ASMR experiences, respectively. The endurance of this pattern highlights the robustness of these findings. Regressions further underscored the power of misophonia, ASMR and musical chills reactions to predict each other. We also found that self-reported misophonia severity was correlated with reactions, valence and arousal responses to misophonia videos, suggesting that misophonia symptoms are related to this real-time task. By contrast, neither general musical sophistication nor specific musical emotional engagement nor musical training had any discernible predictive power for self-reported misophonia, nor misophonia, ASMR or musical chills reactions; moreover, Bayesian analyses provided evidence in favour of null relationships in several of these cases. We conclude that the correlations between the amount of reactions for different types of emotional experiences—both positive (ASMR, musical chills) and negative (misophonia)—may reveal a possible emotional reactivity characteristic in the general population and/or enhanced interoceptive processing.

Findings from prior studies and informal observations are consistent with our suggestion that misophonia, ASMR and musical chills may share common mechanisms. For example, anecdotal evidence abounds that similar types of videos can evoke misophonia in some people and ASMR in other people [66]. ASMR and musical chills are also linked by the anecdotal observation that both involve tingling, suggestive of similar peripheral mechanisms, although this may vary depending on the type of musical chills. In particular, it is possible that ASMR is more linked to social chills (associated with being moved emotionally and in-group bonding) than vigilance chills (associated with a sense of awe or being intimidated) [42], given that ASMR is strongly linked to social closeness [69,100].

Our results add to prior evidence for links between misophonia, ASMR and musical chills. Specifically, the tendency to experience the three emotional phenomena was correlated with overlapping personality characteristics such as neuroticism [37–39] and openness to experience [39,50,61,62]. Furthermore, fMRI studies show that all three experiences co-occur with activity in brain areas such as the insula, ventral striatum and cingulate cortex [17–20,67–71]. The insula is important for both emotion processing and interoceptive processing [74–76], and interoceptive sensitivity predicts ASMR experience [72]. Therefore, our observation that misophonia, ASMR and musical chills reactions are intercorrelated could in principle reflect a general tendency to react emotionally to complex real-world audio-visual stimuli in a manner that is mediated by interoceptive processing of affective states. However, confimation of this would require direct study of interoceptive processing using behavioural or neural measures that can be related to misophonia, ASMR and musical chills. Alternatively, other factors or mechanisms besides emotional reactivity or interoceptive processing, such as a simple bias to respond more, could explain why misophonia, ASMR and musical chills are correlated with each other.

### (a) Limitations and future directions

Our study does have several limitations that point to new studies to perform. First, although we did have attention check and compliance questions throughout the study, we did not use any direct or objective measures of sustained attention or motivation throughout the study, nor did we implement a browser check or headphone requirements to ensure that the sounds were presented exactly as intended. This is a consideration that is becoming increasingly, important especially in auditory research, as investigators move data collection from controlled laboratory environments to online survey formats [81,82].

A second limitation is that because we were attempting to sample from the general population, we did not collect specific or detailed information about other sensory processing and auditory disorders (e.g. autism spectrum disorder, hyperacusis, tinnitus, etc.). It would nevertheless be helpful to track comorbidities across such disorders, for example, anxiety or depression, that could at least partially predict these three experiences.

Third, our reactions task performed online did not allow for the collection of precise timing information about when exactly in the videos the emotional reactions occurred. Future studies should therefore collect such timing information data. This would be important for examining agreement (or disagreement) across participants regarding when emotional reactions occur and whether these reactions are elicited by acoustic features, semantic meaning of particular events and personal memories about those events.

Finally, even though we did collect information about valence and arousal for each video, our reactions task was limited in that it did not allow participants to specify which emotion they were feeling at any given moment. We chose this approach in part because we did not want to bias participants towards negative or positive emotional reactions; however, future research could ask participants to provide information about more nuanced emotions during video viewing. In addition, using a larger set of videos would allow for greater generalizability and for more rigorous investigation of the specific events and acoustic or visual features that drive various emotional reactions.

The finding that self-reported misophonia was correlated with reactions, valence and arousal to misophonia videos supports the possibility that measures derived from real-time misophonia experiences could be useful for clinical purposes in addition to self-report questionnaires. For example, using both retrospective and real-time measures of misophonia could help produce a more robust and complete description of peoples' misophonia for the purpose of diagnosis and evaluation of the efficacy of various types of treatments. Furthermore, real-time measures of misophonia experience might alleviate problems with various inaccuracies in individuals’ reports of their misophonia triggers in everyday life [101–103], although more research would be needed to determine the potential usefulness of such a video task in clinical settings.

Even though we found three kinds of emotional reactions to videos to be correlated with each other, a self-report measure of musical emotional engagement was not correlated with misophonia, ASMR and musical chills reactions to videos. This suggests that the correlations between misophonia, ASMR and musical chills reactions were not related to a general form of high emotional reactivity, but may instead be specific to emotional reactions to complex audio-visual stimuli. Self-reported musical sophistication also was not correlated with A-MISO-S scores, or to misophonia, ASMR, or musical chills reactions. However, because we used exclusively self-report measures of musical sophistication, it is possible that more objective music perception tasks, such as pitch and rhythm discrimination, would be related to misophonia, which could be useful for development of predictive models of misophonia.

### (b) Summary and conclusions

We tested a large sample of participants on self-report measures of misophonia, and real-time reactions to misophonia, ASMR and musical chills videos. We also explored the relationships among misophonia, general musical sophistication and musical emotional engagement. We found that misophonia, ASMR and musical chills reactions to videos were all correlated with each other. Furthermore, misophonia video reactions, valence and arousal were correlated with self-report misophonia scores, suggesting that real-time reactions to videos may have utility for misophonia assessment purposes. Finally, we found no evidence that musical sophistication, musical emotional engagement or musical training were correlated with misophonia. Future studies should more thoroughly test for relations between misophonia and various aspects of musical abilities, including using objective tests because both characteristics involve strong connections between high-level auditory processing and emotion.

## Data Availability

The data are are available at https://osf.io/k6h2m/ [104] and provided in electronic supplementary material [105].

## Endnote

1 This survey was initiated prior to the publication of the consensus definition of misophonia [3] and prior to publication of several validation studies of misophonia assessments [23,24,83].
